# Air- and dustborne fungi in repositories of the National Archive of the Republic of Cuba

**DOI:** 10.15698/mic2022.05.776

**Published:** 2022-04-01

**Authors:** Sofia Borrego, Isbel Vivar, Alian Molina

**Affiliations:** 1 Conservation Preventive Laboratory, National Archive of the Republic of Cuba, Havana, Cuba.

**Keywords:** airborne fungi, dustborne fungi, indoor environment, environmental quality, repositories, archive, biodeterioration

## Abstract

This study has as objectives to determine the concentration and diversity of the air- and dustborne mycobiota in seven National Archive of the Republic of Cuba repositories, and to assess the potential risk of biodeterioration that isolated taxa may have. In the indoor and outdoor environmental microbiological samplings a SAS biocollector was used and the indoor/outdoor (I/O) ratio was determined for each repository. The settled dust was collected during six months. Sørensen's coefficient of similarity (QS) was calculated to compare the isolated taxa among the three studied niches (indoor air, dust, outdoor air). The biodegradation potential of the isolated taxa was determined by semi-quantitative tests. The concentrations in the air of repositories with natural cross-ventilation ranged from 225.2-750.3 CFU m^-3^, while in the Map library with air-conditioning the concentration was significantly lower. The I/O ratios ranged from 0.1-1.7 revealing different environmental qualities. The maximum settled dust load was 22.8 mg/m^2^/day with a top fungal concentration of 6000 CFU g^-1^. 14 and eleven genera were detected in the air and dust respectively with predominance of the genera *Aspergillus, Cladosporium* and *Penicillium*. A QS of 0.8 was obtained between the indoor and the outdoor environments with eleven taxa similar evidencing the incidence of outdoors on the indoor mycobiota. The isolated taxa showed several biodeteriogenic attributes highlighting twelve and 14 taxa from indoor air and dust respectively with positive results for the five tests performed. This demonstrates the potential risk that fungal environmental represent for the preserved documentary heritage.

## INTRODUCTION

In outdoor and indoor environments there is a large number of biotic and abiotic particles of different origin, shape and size suspended in the air, constituting the atmospheric aerosol. They can be classified in different ways considering the origin (biological, organic, inorganic), the location (marine, continental, rural, industrial, urban) and the effect they may have on the surfaces where they settled (chemical, toxic, pathogenic, degrading). Aerosols of biological origin or bioaerosols are particles of microscopic size suspended in the air that can affect human beings causing allergy, toxicity or infection. They can be constituted by protozoa, pollens, microalgae, propagules of multicellular fungi (spores, hyphae, fragments of these and other structures), yeasts (unicellular fungi), bacteria, viruses, toxins and in general any fragment of microorganisms with diverse aerodynamic diameters that comprise dimensions ranging from the order of the nanometers to about 1 mm [1, 2].

Among the components of the atmospheric aerosol, there is also dust. Hence microorganisms can be found freely in the airborne state or associated with dust particles that can settle on the surface of materials [3]. On indoor environments, the air and dust may represent two different niches [4].

Dust is considered an important transport medium for the spread of microorganisms such as bacteria, fungi and spores, as well as minerals and fine particles [5]. This dust can deposit on documents, books, artworks and on many other objects; it varies in quantity and quality depending on the situation of the building, the activities that take place inside, the season of the year, the type of ventilation/air conditioning and the state of conservation of the building [6, 7]. The microbial concentration in the dust reflects the microbial accumulation of a longer period than can be obtained by making a punctual air sampling, which is why it is a matrix that serves as a reservoir of fungal contamination in indoor environments [4, 8]. In this way, when performing the microbial evaluation of the accumulated dust, the behavior of the microbiota during a long period of exposure is being determined. Additionally, dust serves as a source of nutrients for some insects and microorganisms, the latter can also be transported by their particles to the interior of the facilities through ventilation/air-conditioning systems and by people [7, 9]. In archives, libraries and museums, the dust sedimentation can create a microenvironment on the artworks surfaces and the documentary collections that prevents the normal flow of air over them, facilitating the absorption of moisture and favoring the growth and development of microorganisms, mainly of filamentous fungi [10, 11]. Those can generate biofilms on the materials on which they are settled, representing a significant risk of microbial biodeterioration for important documentary collections and art pieces. In this way, the dust constitutes a reservoir of fungal propagules that resupply the air when there is activity in the premises and that can be inhaled [12]. Although fungal propagules are easily dispersed and transported through air, dust, insects, and people [13], fungal growth and colonization on the artwork surfaces and documents found within archives, libraries and museums can also be an important source of air pollution, either with their re-suspended propagules, volatile organic compounds or mycotoxins that are products of their metabolism [4, 9, 14, 15]. For that reason, the assessment of airborne and dustborne fungi provides important information on the mycological quality of indoor environments. Hence, some authors have isolated microorganisms and particularly fungi, both from air and from dust settled in archives, libraries and museums [11, 14, 16–18].

On the other hand, it is known that paper and other documentary materials (microforms, films, audiovisuals, etc.) are an excellent substrate for various heterotrophic organisms, especially fungi, because these materials contain different organic substances such as cellulose or cellulose derivate compounds, glues, inks, pigments and fillers of animal origin [17, 19, 20]. Fungi can easily grow on these materials, biodegrading them [13, 19, 21]. In addition, environmental fungi can be a risk for human health since some species are also potentially pathogenic, causing allergy, infections and toxic effects [9, 22, 23]. The study of indoor microbial contamination in archives, libraries and museums is of great interest to conservators, restorers and researchers due to the impact represented by microorganisms and especially fungi in the deterioration of artifacts and in human health [14, 16, 17, 19, 20, 24–28].

In the National Archive of the Republic of Cuba (NARC) studies have been carried out for years where the fungal concentrations in the air from different repositories have been evaluated [20, 26, 29–33] as well as the presence of fungi on different types of documents made of various materials [20, 24, 34, 35]. However, studies on the fungal characterization of settled dust are very scarce [36, 37]. Therefore, this study has as objectives to determine the concentration and diversity of the air- and dustborne mycobiota in seven NARC repositories, and to assess the potential risk of biodeterioration that isolated taxa may have.

## RESULTS

### Concentration and distribution of airborne fungi in the environment of the analyzed repositories

Fungal concentration obtained in the repositories with natural cross-ventilation ranged between 225.2 colony forming units (CFU) m^−3^ and 750.3 CFU m^−3^, while in the Map library, which has air conditioning, concentration was significantly lower (p ≤ 0.01), i.e., only 43.6 CFU m^−3^. On the other hand, the recorded concentrations in the repositories located on the south side of the building were significantly higher than those obtained in the repositories located on the north side (**Table 1**). When comparing the fungal concentrations obtained per floor, it was observed that the first floor showed the lowest values (R-14 = 370.4 CFU m^−3^ and R-20 = 225.2 CFU m^−3^) while in the second floor the values were highest (R-24 = 750.3 CFU m^−3^ and R-28 = 464.1 CFU m^−3^) followed by the semi-basement (R-4 = 673.2 CFU m^−3^ and R-8 = 415.1 CFU m^−3^). Regarding the environmental quality, it was observed that R-4 and R-24 revealed indoor/outdoor (I/O) ratios ≥ 1.5, i.e. values of 1.5 and 1.7 respectively, indicative of a bad environmental quality, while in the remaining repositories values ≤ 1 were obtained, evidencing a good environmental quality as well as a good ventilation and aeration.

**TABLE 1.** Fungal concentrations (CFU m^-3^) registered in the indoor and outdoor environments and the obtained indoor/outdoor ratios (I/O).

**Natural cross ventilation**												**Air-conditioning environment**		
**Semi-basement**				**1^st^ floor**				**2^nd^ floor**				**1^st^ floor**		
**South**		**North**		**South**		**North**		**South**		**North**		**South**		
**R-4**	**I/O**	**R-8**	**I/O**	**R-14**	**I/O**	**R-20**	**I/O**	**R-24**	**I/O**	**R-28**	**I/O**	**ML**	**I/O**	**Outdoor**
673.2^**a**^	1.5	415.1^**a**^	0.9	370.4^**d**^	0.8	225.2^**c**^	0.5	750.3^**f**^	1.7	464.1^**e**^	1.0	43.6	0.1	450.0

ML: Map library.

a,b,c,d,e,f: Indicative of significant differences when comparing the values obtained in the naturally ventilated repositories located to the south and the north of the building according to the Student's t test (P ≤ 0.01).

**Figure 1:**
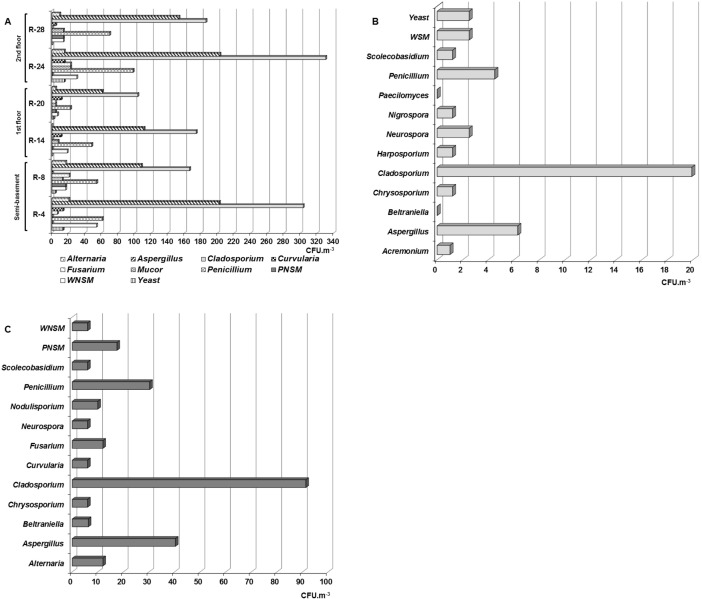
FIGURE 1: Concentrations of the fungal taxa and non-sporulating mycelia isolated from the air of different naturally ventilated repositories (A), the Map library that has an air-conditioned environment (B) and outdoor environment (C). WNSM: white non-sporulating mycelium. PNSM: pigmented non-sporulating mycelium.

A total of seven taxa was detected in the air of the naturally ventilated repositories, predominating genera *Aspergillus* P. Micheli Ex Link, *Cladosporium* Link and *Penicillium* Link, which turned out to be ecologically abundant (**Figure 1A**). However, the genera *Alternaria* Nees, *Curvularia* Boedijn, *Fusarium* Link and *Mucor* Fresen as well as yeasts and two types of non-sporulating mycelia (WNSM: white non-sporulating and PNSM: pigmented non-sporulating) were also detected but at lower concentrations. It should be highlighted that in all the repositories located south of the building, the concentrations of these three predominant genera were higher than those obtained in the repositories located on the north side.

Although the genera *Alternaria, Curvularia* and *Mucor*, as well as the WNSM were detected in low concentrations (≤ 54 CFU m^−3^), they were abundant in the air of the repositories, since their relative frequency (RF) of appearance fluctuated between 83.3 and 100 %, i.e., they were detected in five of the six naturally ventilated repositories. On the other hand, it was found that the *Fusarium* genus and yeasts were common in these environments.

Eleven genera, plus non-sporulating mycelium (WNSM) and yeasts were isolated from the Map library air (**Figure 1B**). The genera were *Acremonium* Link, *Aspergillus, Beltraniella* Subram., *Chrysosporium* Corda, *Cladosporium, Harposporium* Lohde, *Neurospora* Shear & B.O. Dodge, *Nigrospora* Zimm., *Paecilomyces* Bainier, *Penicillium* and *Scolecobasidium* E.V. Abbott. Of them, *Cladosporium* predominated due to its concentration (19.9 CFU m^−3^), followed by *Aspergillus* (6.3 CFU m^−3^) and *Penicillium* (4.5 CFU m^−3^), while the other genera were detected at lower concentrations (1 - 2.5 CFU m^−3^).

From the outdoor air, eleven genera and two non-sporulating mycelia were recorded with a marked predominance of the genus *Cladosporium* (91.3 CFU m^−3^) followed by *Aspergillus* (40.3 CFU m^−3^) and *Penicillium* (30.3 CFU m^−3^; **Figure 1C**). The remaining genera and the non-sporulating mycelia were isolated at lower concentrations (6 - 17.5 CFU m^−3^).

Whereas the genera *Aspergillus, Cladosporium* and *Penicillium* were predominant, it should be noted that the concentrations obtained for *Aspergillus* and *Cladosporium* in most of the naturally ventilated repositories were higher than 100 CFU m^−3^, even in some repositories such as R-4 and R-24 the concentrations were ≥ 200 CFU m^−3^. However, concentrations lower than 160 CFU m^−3^ were obtained for the other genera.

Although most of the studied environments showed typical I/O ratios of good quality environments (I/O ≤ 1), when analyzing the I/O ratio by each predominant genus, the behavior was very different. As shown in **Table 2**, the values of the I/O ratios for the genus *Aspergillus* fluctuated between 1.5 and 5, for the genus *Cladosporium* these values ranged between 1.1 and 3.3, and for the genus *Penicillium* they varied between 0.8 and 3.2. The repositories that showed the highest indexes were R-4 and R-24, while in R-14 and the Map library the ratios were less than 1.

**TABLE 2.** Concentrations and I/O ratio values for the predominant genera in each naturally ventilated repository.

	**R-4**		**R-8**		**R-14**		**R-20**		**R-24**		**R-28**		**Outdoor**	
	**CFU m^−3^**	**I/O**	**CFU m^−3^**	**I/O**	**CFU m^−3^**	**I/O**	**CFU m^−3^**	**I/O**	**CFU m^−3^**	**I/O**	**CFU m^−3^**	**I/O**	**CFU m^−3^**
*Aspergillus*	202	5.0	108	2.7	111	2.8	61	1.5	203	5.0	153	3.8	40.3
*Cladosporium*	303	3.3	166	1.8	174	1.9	104	1.1	330	3.6	186	2.0	91.3
*Penicillium*	61	2.0	54	1.8	48	1.6	23	0.8	98	3.2	70	2.3	30.3

### Species belonging to the predominant genera in the air

Since genera *Aspergillus, Cladosporium* and *Penicillium* were the predominant ones, all isolated strains were identified to species level. In total, 14 species of *Aspergillus*, 14 *Cladosporium* and eleven *Penicillium* were identified (**Table 3**). Several species were abundant and within them were four *Aspergillus* (*A. flavus* Link, *A. niger* Tiegh., *A. terreus* Thom, *A. versicolor* (Vuill.) Tiraboschi), five *Cladosporium* (*C. cladosporioides* (Fresen.) G.A. de Vries, *C. gossypiicola* Pidoplichko & Deniak, *C. lignicola* Link, *C. oxysporum* Berk. & M.A. Curtis, *C. sphaerospermum* Penz.), and four *Penicillium* (*P. chrysogenum* Westling, *P. citrinum* Thom, *P. commune* Thom, *P. simplicissimum* (Oud.) Thom). A total of five species were common (*A. ochraceus* K. Wil., *C. fulvum* Cooke, *C. minourae* Iwatsu, *C. staurophorum* (Kendrick) M.B. Ellis, *C. tenuissimum* Cooke, Grevillea), five were frequent (*A. clavatus* Desm., *A. flavipes* (Bain & Sart) Thom & Church, *A. nidulans* (Eidam) G. Winter, *C. herbarum* (Pers. Fr.) Link, *P. oxalicum* Currie & Thom), three were occasional (*A. oryzae* (Ahlb.) Cahn, *P. aurantiogriseum* Dierckx, *P. griseofulvum* Dierckx) and ten were rare species (*A. athecius* (Raper & Fennell), *A. glaucus* Link, *A. restrictus* G. Smith, *C. allii-porri* (Saccardo & Briard) Boerema, *C. caryigenum* (Ellis & Lang), *C. coralloides* W. Yamamoto, *C. elatum* (Harz) Nannfeldt, *P. corylophilum* Dierckx, *P. digitatum* Saccardo, *P. decumbens* Thom).

**TABLE 3.** Species of the genera *Aspergillus, Cladosporium* and *Penicillium* isolated from indoor air and dust collected in the different repositories analyzed.

**Species**	**Semibasement**		**1^st^ floor**		**2^nd^ floor**		**1^st^ floor**			
	**South**	**North**	**South**	**North**	**South**	**North**	**South**			
	**R-4**	**R-8**	**R-14**	**R-20**	**R-24**	**R-28**	**ML**			
	**RD (%)**							**RF (%)**	**EC**	**Outdoor**
**AIR**										
*Aspergillus athecius* (Raper & Fennell)	0	0	0	0	0	0	2.8	14.3	R	0
*A. clavatus* Desm.	0	0	10.2	0	1.6	6.1	0	42.9	F	0
*A. flavipes* (Bain & Sart) Thom & Church	0	0	8.4	2.0	3.2	9.4	0	57.1	F	0
*A. flavus* Link	19.1	8.0	19.6	8.2	14.8	6.2	11.7	100	A	5.2
*A. glaucus* Link	0	0	0	0	0	0	5.5	14.3	R	0
*A. nidulans* (Eidam) G. Winter	7.3	1.0	5.2	0	3.2	0	0	57.1	F	0
*A. niger* Tiegh.	10.3	12.0	12.3	7.0	6.4	8.1	8.5	100	A	3.2
*A. ochraceus* K. Wil.	2.5	1.0	3.1	3.1	0	3.2	0	71.4	C	2.1
*A. oryzae* (Ahlb.) Cahn	3.0	0	5.4	0	0	0	0	28.6	O	1.1
*A. parasiticus* Speare	0	0	0	0	0	0	0	0	-	1.3
*A. penicillioides* Spegazzini	0	0	0	0	0	0	0	0	-	2.1
*A. restrictus* G. Smith	0	0	0	0	0	0	2.7	14.3	R	0
*A. terreus* Thom	6.4	1.0	3.0	2.1	2.1	1.0	0	85.7	A	1.1
*A. versicolor* (Vuill.) Tiraboschi	1.3	2.0	5.5	1.5	2.1	1.0	0	85.7	A	0
*Cladosporium allii-porri* (Saccardo & Briard) Boerema	0	0	0	0	0	0	2.7	14.3	R	0
*C. caryigenum* (Ellis & Lang)	0	0	0	0	0	0	8.7	14.3	R	0
*C. cladosporioides* (Fresen.) G.A. de Vries	11.9	18.2	20.3	20.0	21.0	19.0	8.7	100	A	0
*C. coralloides* W. Yamamoto	0	0	0	0	0	0	8.7	14.3	R	12.2
*C. elatum* (Harz) Nannfeldt	0	0	0	0	0	0	2.8	14.3	R	6.8
*C. fulvum* Cooke	12.0	1.0	6.8	5.0	0	3.0	0	71.4	C	2.4
*C. gossypiicola* Pidoplichko & Deniak	2.4	0	3.0	5.0	5.0	3.0	0	85.7	A	0
*C. herbarum* (Pers. Fr.) Link	6.0	3.3	0	7.2	0	11.5	0	57.1	F	0
*C. lignicola* Link	8.2	6.3	5.8	2.0	3.0	0	2.8	85.7	A	2.8
*C. minourae* Iwatsu	2.5	0	1.1	0	2.4	1.1	2.8	71.4	C	2.5
*C. oxysporum* Berk. & M.A. Curtis	2.1	5.4	0	7.4	1.1	7.9	0	85.7	A	5.6
*C. sphaerospermum* Penz.	5.4	6.8	10.0	6.7	6.3	7.8	0	85.7	A	2.8
*C. staurophorum* (Kendrick) M.B. Ellis	0	4.5	0	5.0	3.0	5.0	2.8	71.4	C	0
*C. tenuissimum* Cooke, Grevillea	1.3	1.1	0	2.3	0	1.1	2.8	71.4	C	1.4
*Penicillium aurantiogriseum* Dierckx	0	1.0	2.0	0	0	0	0	28.6	O	1.2
*P. brevicompactum* Dierckx	0	0	0	0	0	0	0	0	-	1.1
*P. chrysogenum* Westling	5.0	3.0	4.0	6.0	9.0	8.0	0	85.7	A	4.5
*P. citrinum* Thom	1.0	5.0	3.0	1.0	0	2.0	10.3	85.7	A	4.2
*P. commune* Thom	3.0	2.0	3.0	3.0	4.0	4.0	0	85.7	A	0
*P. corylophilum* Dierckx	0	0	0	0	0	0	4.7	14.3	R	1.1
*P. decumbens* Thom	0	1.0	0	0	0	0	0	14.3	R	0
*P. digitatum* Saccardo	0	0	0	0	0	1.0	0	14.3	R	0
*P. griseofulvum* Dierckx	0	1.0	1.0	0	0	0	0	28.6	O	0
*P. oxalicum* Currie & Thom	1.1	0	1.1	0	3.1	0	0	42.9	F	0
*P. simplicissimum* (Oud.) Thom	1.2	5.1	1.1	3.3	1.1	4.9	0	85.7	A	0
**DUST**										
*Aspergillus chevalieri* L. Mangin	6*	7*	8*	2	2	3	16*	100	A	-
*A. clavatus* Desm.	0	0	0	0	3	3	0	28.6	O	-
*A. flavus* Link	4	9	5	4	3	5	12	100	A	-
*A. glaucus* Link	3	3	1	3	1	2	0	85.7	A	-
*A. niger* Tiegh.	5	6	5	6	4	7	17	100	A	-
*A. oryzae* (Ahlb.) Cahn	3	2	2	2	3	0	0	71.4	C	-
*A. penicillioides* Spegazzini	2	3	4	0	1	0	0	57.1	F	-
*A. restrictus* G. Smith	10	2	2	3	3	3	0	85.7	A	-
*A. terreus* Thom	0	2	0	2	0	0	0	28.6	O	-
*A. versicolor* (Vuill.) Tiraboschi	0	2	2	3	2	2	0	71.4	C	-
*C. basiinflatum* Bensch, Crous & U. Braun	0	5	0	3	0	5	0	42.9	F	-
*C. caryigenum* (Ellis & Lang)	0	0	0	0	0	0	6	14.3	R	-
*C. cladosporioides* (Fresen.) G.A. de Vries	12	9	14	12	10	9	14	100	A	-
*C. elatum* (Harz) Nannfeldt	4	0	3	1	2	0	0	57.1	F	-
*C. herbarum* (Pers. Fr.) Link	7	5	10	2	4	3	0	85.7	A	-
*C. hillianum* Bensch, Crous & U. Braun	0	4	0	3	0	4	0	42.9	F	-
*C. oxysporum* Berk. & M.A. Curtis	8	6	3	4	6	4	0	85.7	A	-
*C. sphaerospermum* Penz.	6	4	4	10	6	6	0	85.7	A	-
*C. tenuissimum* Cooke, Grevillea	3	7	7	5	5	9	0	85.7	A	-
*Penicillium aurantiogriseum* Dierckx	0	2	0	0	0	3	0	28.6	O	-
*P. chrysogenum* Westling	3	3	3	8	2	5	8	100	A	-
*P. citrinum* Thom	4	5	3	6	4	2	5	100	A	-
*P. commune* Thom	0	3	2	2	0	3	0	57.1	F	-
*P. decumbens* Thom	0	2	0	0	2	0	0	28.6	O	-
*P. digitatum* Saccardo	0	3	0	0	0	2	0	28.6	O	-
*P. janczewskii* K.M. Zalessky	2	0	2	0	1	0	13	57.1	F	-
*P. simplicissimum* (Oud.) Thom	2	2	2	2	3	3	2	100	A	-

RF: Relative frequency. EC: Ecological categories [30] are Abundant taxa (A) when RF = 100 - 81%, Common taxa (C) when RF = 80 - 61%, Frequent taxa (F) when RF = 60 - 41%, Occasional taxa (O) when RF = 40 - 21% and Rare taxa (R) when RF = 20 - 0.1%.

*: Indicates the existence of different percentages of teleomorphs (2-10%).

17 species isolated from the outdoor environment were also detected in some of the indoor environments and those were *A. flavus, A. niger, A. ochraceus, A. oryzae, A. terreus, C. coralloides, C. elatum, C. fulvum, C. lignicola, C. minourae, C. oxysporum, C. sphaerospermum, C. tenuissimum, P. aurantiogriseum, P. chrysogenum, P. citrinum, P. corylophilum* (**Table 3**).

### Characterization of the settled dust

The settled dust load in the repositories showed values that ranged between 3.9 and 22.8 mg/m^2^/day (**Table 4**). It was observed that the repositories located in the semi-basement of the building (R-4 and R-8) had dust values that differed significantly, with R-8 (situated on the north side) revealing the highest accumulation of dust. The largest dust amounts were accumulated in R-14 located on the south side of the first floor and in R-24 set on the north part of the second floor. On the other hand, fungal concentrations that fluctuated between 1.2 x 10^2^ and 6.0 x 10^3^ CFU g^−1^ of dust were obtained. But the repositories set on the south side of the building showed significantly higher loads than those situated on the north part, with the highest concentrations being detected in the repositories on the second floor and in the Map library, which is a climate-controlled repository.

**TABLE 4.** Total load of dust collected in the studied repositories and fungal concentrations detected in them.

**Repository**	**Location in the building**		**Total load of collected dust (mg/m^2^/days)**	**Fungal concentrations (CFU g^−1^)**
R-4	Semi-basement	South	4.0^**a**^	5.5 × 10^2 **d**^
R-8		North	9.3^**b**^	4.1 × 10^2 **c**^
R-14	1^st^ floor	South	22.8^**d**^	3.0 × 10^2 **b**^
R-20		North	4.8^**a**^	1.2 × 10^2 **a**^
R-24	2^nd^ floor	South	3.9^**a**^	4.2 × 10^3 **f**^
R-28		North	13.7^**c**^	2.7 × 10^3 **e**^
Map library	1^st^ floor	South	8.5^**b**^	6.0 × 10^3 **g**^

a,b,c,d,e,f,g: Indicates significant differences according to LSD test (P ≤ 0.05) when comparing each determination among the different repositories. Each determination was made at 5 points in each repository and the data were averaged (n = 5).

**Figure 2:**
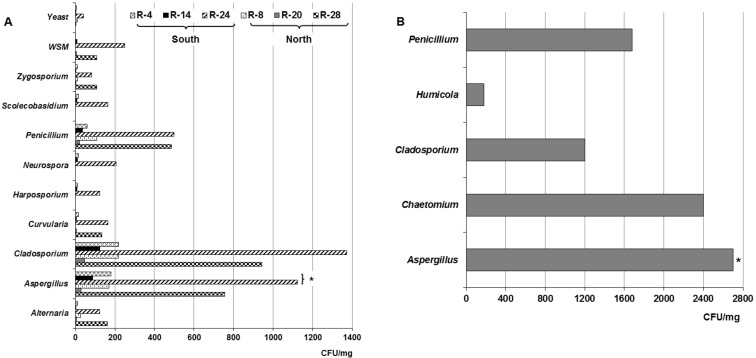
FIGURE 2: Concentrations of the fungal taxa, non-sporulating mycelium and yeast detected in the dust collected from the repositories with natural cross-ventilation system (A) and the Map library with air-conditioning (B). WNSM: White non-sporulating mycelium. *: Indicates different concentrations of teleomorph detected in the naturally ventilated repositories and Map library, all situated to the south part of the building.

**Figure 2** shows the different isolated genera from dust collected both in the naturally ventilated repositories as well as in the Map library, a climate-controlled repository. As observed in the naturally ventilated repositories, nine genera, a non-sporulating mycelium (WNSM) and yeasts were obtained, while five taxa were isolated in the Map library. The genera *Alternaria, Aspergillus, Cladosporium, Penicillium* and *Zygosporium* Mont. were isolated from all ventilated repositories. Instead the genera *Curvularia, Harposporium, Neurospora* and *Scolecobasidium* were only isolated from the dust collected in R-4, R-14 and R-24, repositories located on the south side of the building. Some *Aspergillus* teleomorph were detected in the dust collected only in the repositories situated in the southern part of the building (R-4, R-14, R-24 and Map library). A WNSM was isolated from the dust collected in R-14, R-20, R-24 and R-28, while yeasts were detected in five of the repositories, except in R-28. In this case again, the predominant genera were *Aspergillus, Cladosporium* and *Penicillium*. In the Map library the predominant genera were also *Aspergillus* (2700 CFU m^−3^), *Cladosporium* (1200 CFU m^−3^) and *Penicillium* (1680 CFU m^−3^), although the genera *Chaetomium* Kunze and *Humicola* Traaen were also detected at lower concentrations.

### Species characterization in the dust

Fungal strains belonging to predominant genera *Aspergillus, Cladosporium* and *Penicillium* were identified to species level. Totally ten species belonging to *Aspergillus*, nine to *Cladosporium* and eight to *Penicillium* were identified (**Table 3**). According to the percentage of the RF, 13 species were found to be abundant and were *A. chevalieri* L. Mangin, *A. flavus, A. glaucus, A. niger, A. restrictus, C. cladosporioides, C. herbarum, C. oxysporum, C. sphaerospermum, C. tenuissimum, P. chrysogenum, P. citrinum, P. simplicissimum*. Although the species *A. chevalieri, A. flavus, A. niger, C. cladosporioides, P. chrysogenum, P. citrinum* and *P. simplicissimum* showed RF = 100%, i.e., they were detected in all repositories, their relative densities (RD) were different. In this case, *C. cladosporioides* showed an RD ≤ 14% in all repositories (equivalent to ≤ 17 CFU g^−1^ in ventilated repositories and 1200 CFU g^−1^ in Map library), followed by *A. chevalieri, A. flavus* and *A. niger* (RD = 3 - 17%) as well as *P. chrysogenum* and *P. citrinum* (RD = 3 - 8%), while *P. simplicissimum* showed a RD between 2 and 3%. In the Map library, the percentages of the three *Aspergillus* species mentioned above represented high concentrations (324 - 459 CFU g^−1^) because this genus was found at a very high concentration in the collected dust (2700 CFU g^−1^); the same happened with the mentioned *Penicillium* species, the concentrations in the dust fluctuated from 34 to 134 CFU g^−1^ because this genus was found at a concentration of 1680 CFU g^−1^. The other abundant species were detected at RF = 85.7%, i.e., in four of the five repositories (*A. glaucus, A. restrictus, C. herbarum, C. oxysporum, C. sphaerospermum, C. tenuissimum*), and their RD were also different but also low (equivalent to < 20 CFU g^−1^). The species *C. sphaerospermum, C. tenuissimum* and *C. oxysporum* presented the highest RD (RD = 3 - 10%), while *A. restrictus* and *A. glaucus* showed the lowest RD (RD = 1–3%), with the exception of *A. restrictus* that revealed an RD = 10% only in the dust collected in R-4. Two species were classified as common with a RF of 71.4% (*A. oryzae* and *A. versicolor*), but their RDs varied between 2 and 3%, i.e., percentages and concentrations low. Six species were frequent (*A. penicillioides* Spegazzini, *C. basiinflatum* Bensch, Crous & U. Braun, *C. elatum, C. hillianum* Bensch, Crous & U. Braun, *P. commune* and *P. janczewskii* KM Zalessky), but only *C. basiinflatum* showed a RD between 3 and 5% (equivalent to < 20 CFU g^−1^), the other frequent species showed smaller RD (≤ 3%), with the exception of *A. penicillioides* in R-14 (RD = 4%, equivalent to 13 CFU m^−3^) and *P. janczewskii* in Map library (RD = 13%, equivalent to 218 CFU g^−1^). Five species were classified as occasional (*A. clavatus, A. terreus, P. aurantiogriseum, P. decumbens* and *P. digitatum*) and all showed a low RD (≤ 3%). Only one species was found to be rare (*C. caryigenum*) and was isolated from the Map library with an RD of 6%, equivalent to 72 CFU g^−1^.

### Similarity of the different taxa obtained in air and dust

Comparisons were made between the three niches studied (indoor air, outdoor air, and dust collected indoors). Taxa isolated from indoor air were compared with those obtained from outdoor air. Taxa from indoor air were compared with those in collected dust. Taxa detected in outdoor air were likened with those in collected dust (**Table 5**). When the isolated taxa from the indoor environment were compared with those detected in the outdoor air a very high Sørensen's coefficient of similarity (QS) was obtained (QS of 0.8). In that case, nine taxa were similar between both environments and they were *Alternaria, Aspergillus, Chrysosporium, Cladosporium, Curvularia, Fusarium, Neurospora, Penicillium* and *Scolecobasidium*. The QS of *Aspergillus, Cladosporium* and *Penicillium* species between these two environments were 0.7, 0.8 and 0.3, respectively, showing that the greatest similarities were between *Cladosporium* and *Aspergillus*. The species found both indoors and outdoors were *C. cladosporioides, C. oxysporum, C. tenuissimum, C. sphaerospermum, C. elatum, C. fulvum, C. lignicola, C. minourae, A. flavus, A. niger, A. ochraceus, A. oryzae, A. parasiticus, A. penicillioides* and *A. terreus.*

**TABLE 5.** Sørensen's coefficient of similarity (QS) obtained by comparing the total number of taxa and species belonging to *Aspergillus, Cladosporium* and *Penicillium* genera isolated in the three studied niches.

**Relationship of taxa or species between:**	**QS_TOTAL_**	**QSA_*spergillus* species_**	**QS_*Cladosporium* species_**	**QS_*Penicillium* species_**
Indoor environments and outdoor environment	0.8	0.7	0.8	0.3
Outdoor environment and dust	0.6	0.6	0.6	0.5
Indoor environments and dust	0.7	0.8	0.6	0.7

The isolated taxa in the indoor air and dust showed a QS of 0.7, revealing a high similarity. Eight taxa were found to be similar between both niches and they were *Alter-naria, Aspergillus, Cladosporium, Curvularia, Harposporium, Neurospora, Penicillium* and *Scolecobasidium*. Regarding *Aspergillus, Cladosporium* and *Penicillium* species, the QS obtained were 0.8, 0.6 and 0.7, respectively. The commoly isolated species were *A. clavatus, A. flavus, A. glaucus, A. niger, A. oryzae, A. penicillioides, A. restrictus, A. terreus, A. versicolor, C. caryigenum, C. cladosporioides, C. elatum, C. herbarum, C. oxysporum, C. sphaerospermum, C. tenuissimum, P. aurantiogriseum, P. chrysogenum, P. citrinum, P. commune, P. decumbens, P. digitatum* and *P. simplicissimum.*

Taxa similarity between the outdoor environment and dust yielded a QS of 0.6; this coefficient shows a moderate similarity with seven similar genera (*Alternaria, Aspergillus, Cladosporium, Curvularia, Neurospora, Penicillium* and *Scolecobasidium*). However, when comparing the taxa isolated in these two niches with those detected in the indoor air of the repositories, it is noted that there was a similarity in seven of them too (*Alternaria, Aspergillus, Cladosporium, Curvularia, Neurospora, Penicillium, Scolecobasidium*). QS obtained for *Aspergillus, Cladosporium* and *Penicillium* species between outdoor air and dust were 0.6, 0.6 and 0.5, respectively, showing a moderate similarity. *Aspergillus flavus, A. niger, A. oryzae, A. penicillioides, A. terreus, C. cladosporioides, C. oxysporum, C. tenuissimum, C. sphaerospermum, C. elatum, P. aurantiogriseum, P. citrinum* and *P. chrysogenum* were found in indoor dust and in outdoor air. When comparing these species with those obtained in the indoor air of the repositories, it can be seen that all of them are similar.

### Characterization of the biodeteriogenic potential

The biodegradative activities were only determined for the predominant genera species (*Aspergillus, Cladosporium* and *Penicillium*) that were isolated from indoor air and dust in the studied repositories (**Table 6**). Of them, ten strains isolated from air (27%) showed high CEI values (IE ≥ 0.7), but only *A. niger* 1, *A. flavus* 1 and *P. chrysogenum* 1 exhibited the highest IE (> 0.75). The rest of the strains had the following behavior: 14 strains (37.8%) exhibited a moderate CEI and twelve strains (32.4%) showed a low CEI; only *C. oxysporum* 1 was not able to degrade cellulose. It is necessary to note that although 75% of the *Aspergillus* strains exhibited a moderate to high activity, the *Penicillium* strains revealed a higher ability (90.9%).

**TABLE 6.** Enzyme Index (EI) obtained of *Aspergillus, Cladosporium* and *Penicillium* strains isolated from the indoor air and collected dust from NARC repositories.

**Species**	**Cellulolytic Activity CEI**	**Amylolytic Activity AEI**	**Proteolytic Activity PEI**	**Acids production (pH)**	Pigment Excretion*
**AIR**					
*Aspergillus athecius*	0.62	0	0	5.90	-
*Aspergillus clavatus* 1	0.71	0.62	0.58	6.07	-
*Aspergillus flavipes*	0.60	0.63	0.58	3.72	+ (yellow)
*Aspergillus flavus* 1	0.76	0.74	0.74	6.22	-
*Aspergillus glaucus* 1	0.66	0.59	0.75	6.60	-
*Aspergillus nidulans*	0.51	0.50	0.52	3.17	-
*Aspergillus niger* 1	0.78	0.71	0.73	5.41	-
*Aspergillus ochraceus* 1	0.72	0.75	0.74	5.40	+ (brown)
*Aspergillus oryzae* 1	0.65	0.68	0.71	4.33	+ (brown dark)
*Aspergillus restrictus* 1	0.57	0.56	0.59	5.82	-
*Aspergillus terreus* 1	0.52	0.55	0.53	4.82	+ (brown)
*Aspergillus versicolor* 1	0.60	0.68	0.62	4.13	-
*Cladosporium allii-porri*	0.51	0.65	0	6.55	-
*Cladosporium caryigenum* 1	0.70	0	0	6.25	+ (green olive)
*Cladosporium cladosporioides* 1	0.66	0.58	0.70	3.34	+ (green dark)
*Cladosporium coralloides*	0.58	0.55	0	5.85	+ (brown)
*Cladosporium elatum* 1	0.56	0.61	0.68	5.66	+ (brown)
*Cladosporium fulvum*	0.50	0	0.62	6.32	-
*Cladosporium gossypiicola*	0.65	0.68	0.56	5.72	+ (green dark)
*Cladosporium herbarum* 1	0.68	0.72	0.62	6.50	+ (green dark)
*Cladosporium lignicola*	0.52	0.58	0.60	6.60	+ (brown)
*Cladosporium minourae*	0.58	0.61	0.69	5.11	-
*Cladosporium oxysporum* 1	0	0.59	0.70	6.02	+ (brown)
*Cladosporium sphaerospermum* 1	0.66	0.54	0	6.30	+ (green dark)
*Cladosporium staurophorum*	0.56	0.62	0	6.30	+ (brown)
*Cladosporium tenuissimum* 1	0.52	0.63	0.54	6.11	+ (brown)
*Penicillium aurantiogriseum* 1	0.65	0.62	0.69	5.21	+ (yellow)
*Penicillium brevicompactum*	0.62	0.58	0.67	4.51	-
*Penicillium chrysogenum* 1	0.78	0.69	0.74	4.80	-
*Penicillium citrinum* 1	0.72	0	0.61	5.27	+ (yellow)
*Penicillium commune* 1	0.62	0.54	0.64	6.01	-
*Penicillium corylophilum*	0.67	0.68	0.69	3.07	+ (green dark)
*Penicillium decumbens* 1	0.72	0.65	0.58	5.35	-
*Penicillium digitatum* 1	0.58	0.62	0.67	5.19	-
*Penicillium griseofulvum*	0.71	0.69	0.62	5.15	-
*Penicillium oxalicum*	0.62	0.57	0.72	3.21	-
*Penicillium simplicissimum* 1	0.71	0.68	0.58	6.60	-
**DUST**					
*Aspergillus chevalieri*	0.72	0.65	0.68	4.46	-
*Aspergillus clavatus* 2	0.61	0.68	0.68	5.99	-
*Aspergillus flavus* 2	0.63	0.71	0.56	4.62	-
*Aspergillus glaucus* 2	0.54	0.56	0.63	6.70	+ (yellow)
*Aspergillus niger* 2	0.78	0.72	0.76	5.00	-
*Aspergillus oryzae* 2	0.76	0.70	0.71	4.13	+ (brown dark)
*Aspergillus penicillioides* 2	0.66	0.54	0.57	6.12	-
*Aspergillus restrictus* 2	0.50	0.50	0.59	6.15	-
*Aspergillus terreus* 2	0.52	0.55	0.60	5.19	+ (brown)
*Aspergillus versicolor* 2	0.71	0.72	0.65	5.21	+ (yellow)
*Cladosporium basiinflatum*	0.73	0.71	0.75	5.17	+ (brown)
*Cladosporium caryigenum* 2	0.56	0.58	0.67	5.29	+ (green olive)
*Cladosporium cladosporioides* 2	0.54	0.60	0.70	5.26	+ (green dark)
*Cladosporium elatum* 2	0.50	0.50	0.56	6.31	-
*Cladosporium herbarum* 2	0.62	0.51	0.52	5.41	-
*Cladosporium hillianum*	0.58	0.53	0.54	4.16	+ (amber dark)
*Cladosporium oxysporum* 2	0.68	0.65	0.52	6.62	+ (brown dark)
*Cladosporium sphaerospermum* 2	0.65	0.50	0.51	6.30	+ (brown dark)
*Cladosporium tenuissimum* 2	0.57	0.65	0.67	5.71	-
*Penicillium aurantiogriseum* 2	0.57	0	0.65	5.20	+ (yellow)
*Penicillium chrysogenum* 2	0.59	0.66	0.71	4.45	-
*Penicillium citrinum* 2	0.73	0.72	0.70	4.36	+ (yellow)
*Penicillium commune* 2	0.63	0.52	0	5.05	-
*Penicillium decumbens* 2	0.70	0.72	0.75	5.08	+ (yellow)
*Penicillium digitatum* 2	0.58	0.61	0.70	4.15	+ (amber)
*Penicillium janczewskii*	0.62	0.59	0.68	3.47	+ (yellow)
*Penicillium simplicissimum* 2	0.71	0.70	0.75	6.61	-

CEI: Cellulolytic Enzymatic Index. AEI: Amylolytic Enzymatic Index. PEI: Proteolytic Enzymatic Index. Enzymatic index (EI) = 0.5 - 0.59 is low, EI = 0.6 - 0.69 is moderate, EI ≥ 0.7 is high. +: Indicates excretion of pigments. -: Indicates no excretion of pigment. pH values < 7 reveal acid production.

* These pigments were detected in CMC medium and a culture medium with similar composition to CMC but with glucose (1%) as control.

Regarding the amylolytic activity, the majority of the strains (78.4%) showed a moderate or low activity; only four strains evidenced a high activity (*A. flavus* 1, *A. niger* 1, *A. ochraceus* 1, *C. herbarum* 1) and other four did not degrade the starch. Likewise, 59.5% of the strains degraded gelatin moderately or with a low EI (PEI ≤ 0.69) and only nine strains (24.3%) showed the highest PEI (*A. flavus* 1, *A. glaucus* 1, *A. niger* 1, *A. nidulans, A. ochraceus* 1, *A. oryzae* 1, *C. oxysporum* 1, *P. chrysogenum* 1, *P. oxalicum*) while six strains (16.2%) did not showed activity. Although acids were excreted by all the taxa, ten of them (27%) should be highlighted for having decreased the pH of the culture medium to values below 5 (*A. flavipes, A. nidulans, A. oryzae* 1, *A. terreus* 1, *A. versicolor* 1, *C. cladosporioides* 1, *P. brevicompactum, P. chrysogenum* 1, *P. corylophilum, P. oxalicum*); whereas, 18 strains excreted different pigments (48.6%) with the prevalence of yellow, green dark and brown colors.

Also, all the species isolated from the dust were able to degrade cellulose with different intensity. Eight strains revealed very high enzyme indices (EI ≥ 0.7), but among them two strains stood out (*A. niger* 2, *A. oryzae* 2) since their CEI were the highest (EI > 0.75).

In relation to the number of strains detected on indoor air by genus with moderate or high CEI, it was obtained that *Penicillium* showed ten strains (27%), followed by *Aspergillus* (nine strains, 24.3%) and *Cladosporium* (5 strains, 13.5%). However, the strains detected in the dust had a different behavior, since *Aspergillus* showed the highest strains number (seven) with a moderate or high CEI (25.9%) followed by *Penicillium* (five strains, 18.5%) and *Cladosporium* (four strains, 14.8%).

Of the total strains detected in the dust, 29.6% were able to express the highest amylolytic indexes (AEI ≥ 0.7), while the 25.9% of them exhibited a moderate AEI with a slight prevalence of *Cladosporium* species (11.1% of *Cladosporium* species vs. 7.4% of *Aspergillus* and *Penicillium* species); but one strain did not show amylolytic activity (*P. aurantiogriseum* 2). In relation to the proteolytic activity, 18 strains (66.7%) showed a moderate or high PEI, highlighting four of them (*A. niger* 2, *C. basiinflatum, P. decumbens* 2, *P. simplicissimum* 2) while only one strain did not degrade proteins (*P. commune* 2). Although all strains excreted acids, eight of them (29.6%) caused a marked decrease in pH (pH < 5); therefore, they were the biggest producers of acids while 15 strains (55.6%) excreted pigments with a prevalence of yellow and brown colors.

As can be seen in **Table 6**, of indoor air, twelve strains (32.4%) showed positive results in the five tests performed (cellulolytic, amylolytic, and proteolytic activity, as well as excretion of acids and pigments), 21 of them (56.8%) were positive in four of the five tests, three strains (8.1%) were positive in three of the tests, while only one strain (2.7%) showed positivity in two of the analyzes performed. Regarding the detected strains in the dust, it was obtained that 14 of them (51.9%) gave positive results to the five tests, twelve strains (44.4%) were positive in four analyzes and only one strain (3.7%) was positive in three assays. It should be noted that of the isolated strains from both indoor air and dust, it was obtained that nine of them (three from air and six from dust) showed the highest risk for the preserved documents, since they displayed the greatest degrading ability (*A. flavus* 1, *A. niger* 1, *A. ochraceus* 1, *A. niger* 2, *A. oryzae* 2, *C. basiinflatum, P. citrinum* 2, *P. decumbens* 2, *P. simplicissimum* 2).

## DISCUSSION

Studies of airborne fungi in indoor environments always reveal the taxa diversity in the air. But airborne microorganisms, particularly fungi, do not remain suspended in the air indefinitely. They settle at some time and, under favorable conditions, may grow on the surfaces where they have settled. Therefore, airborne fungi are a potential risk factor for biodeterioration [3, 11]. The same happens with dust, which, when settling on objects and documents, and under favorable conditions of temperature (T) and water activity (a_w_), can favor the growth of fungi [6, 11].

For years, NARC has performed fungal concentration determinations in the indoor environments of their repositories [30, 31, 33, 34, 36]. But many of these studies were made using a sedimentation method whose results are not comparable because as it is not quantitative. However, in other investigations where a biocollector was used, the reported total concentrations of fungi were similar to those obtained in this study. Borrego and Perdomo [29] carried out a study in six repositories with natural cross-ventilation systems and reported fungal concentrations that ranged between 60 and 550 CFU m^−3^. Later in a similar study [32] the referred concentrations ranged between 92 CFU m^−3^ and 785 CFU m^−3^. Likewise, these data were similar and/or lower than those obtained by other authors [28, 37]. On the other hand, previous studies performed in the Map library of NARC report concentrations of 38 CFU m^−3^ [20] and 40.8 CFU m^−3^ [26].

All these total concentrations of fungi in air showed a tendency to be higher in the repositories located in the semi-basement and the second floor of the building and to be lower in those repositories located on the first floor. This pattern was similar to a previous report [32]. If the indoor environment is influenced by the outdoors [4, 14, 32, 38–40], then it would be expected that the concentration should decrease with the increase in height, a matter that did not occur. This was possibly due to the fact that the building, due to its location within the city, has been constantly influenced by high pollution [41] and a high movement of bioaerosols. This may be due to the interrelation of two fundamental aspects. The first is justified by the size of the fungal spores. Fungi with small spores were detected in the outdoor environment of the archive building (e.g., *Aspergillus, Penicillium, Cladosporium, Chrysosporium*, etc.), and it is known that smaller spores are dominant at higher altitudes, while larger spores and conidia are more frequent at lower levels [42, 43]. The second is associated with the high vehicular and pedestrian traffic that occurs in the streets surrounding the archive, which together with the wind generate turbulence of dust and bioaerosols (harbouring small fungal spores) that impact the building, facilitating their entry to the repositories through the ventilation ducts. This phenomenon is favored by the large number of gardens with trees in the surroundings of the building [4].

When the obtained fungal concentrations were compared with the threshold limits reported by Roussel *et al.* [16], it was evidenced that R-4 and R-24 had environments with high pollution since the concentrations were between 560 and 1000 CFU m^−3^, while the rest of the repositories showed environments with low to moderate pollution. This behavior coincides with the limit established by the Portuguese legislation of 500 CFU m^−3^ for fungi [18], when the results were compared.

Nevertheless, it is suggested that the outdoor airborne fungi concentration is generally higher than that in the indoor environment, as the outdoor environment is a significant modulator of fungal concentrations in naturally ventilated indoor environments [11, 28]; hence, it has been reported that the I/O ratio is an indicator of microorganism emission. If this ratio is ≤ 1, the outdoor environment is the main source of bioparticle emission into the indoor environments [11, 44], but if the I/O ratio is higher than 1 then there are indoor sources of contamination [11, 28, 44]. According to these criteria, variations in the pollution were observed. In R-4 and R-24 the calculated I/O ratios revealed values of 1.5 and 1.7, typical of bad environmental quality, while the rest of the repositories presented I/O ratio values lower than 1, which shows good environmental quality. It is possible that in R-4, because it is located in the semi-basement, there is a lack of air circulation and this caused conditions that favored the appearance of amplification areas, stimulating an increase in the fungal concentration inside. But in the case of R-24 it is possible that two aspects are taking place at the same time. The first is related to the ducts that are located in about 4.5 m height. As these ducts are rarely cleaned, it is very likely that they are contributing to the continuous introduction of fungal propagules into the repository air; while the second aspect is linked to the bioparticles that remain attached to the repository walls which are not very smooth. It is possible that these particles, together with the T and relative humidity (RH) conditions existing in the repository, are causing the fungal growth not visible to the naked eye in some areas of the walls, when they are impacted by the air they release propagules to the indoor, increasing the environmental fungal load and, hence, the I/O ratio. About the documentation preserved in both repositories, in systematic reviews made by the conservators and researchers, fungal growth has not been detected in them. Therefore, apparently it is not the documents that are contributing to the high I/O ratio. However, it is important to perform systematic fungal isolations from the documents to prove this hypothesis.

From the indoor air of the naturally ventilated repositories, a total of seven genera, two non-sporulating mycelia and yeasts were isolated, while from Map library eleven genera, a non-sporulating mycelium and yeasts were detected, with a marked prevalence of the genera *Aspergillus, Cladosporium* and *Penicillium* in all of them. Similar results were obtained in previous studies carried out at the NARC [20, 26, 29, 30, 32–34] and in other Cuban archives and museums [45–51]. These genera are also very common in the NARC outdoor air [52]. However, *Acremonium* and *Beltraniella* that were first isolated in the Map library environment showed a low ecological impact, turning out to be rare genera. In some previous studies performed in NARC, in other Cuban archives, as well as in archives, library and museums of other countries, findings of non-sporulating mycelia have been reported, referring mainly to two of them, one that is white to the naked eye (WNSM) and the other that is pigmented (PNSM) [20, 26, 44–46, 53]. These mycelia have been detected in different concentrations and are still being isolated from the environments of the NARC repositories as shown in this study.

When comparing the taxa obtained in the indoor air with those the outdoor one, it was observed that ten taxa were detected in these environments for a 66.7% of coincidence. The matching genera in both environments were *Alternaria, Aspergillus, Beltraniella, Chrysosporium, Cladosporium, Curvularia, Fusarium, Neurospora, Penicillium* and *Scolecobasidium*. It should be noted that most of these taxa have been reported by other authors in archives, libraries and museums in various countries [28, 37, 38, 48, 54]. These results show that there is a high degree of coincidence in the fungal diversity between the indoor and outdoor environments, evidencing the high influence of the outdoor environment on the mycobiota of indoors, an aspect attributable to the existence of natural ventilation in most of the repositories.

Regarding the I/O ratios of the predominant genera *Aspergillus, Cladosporium* and *Penicillium*, high values were obtained in most of the repositories (1.8 - 5.0), suggesting that several problems are occurring: a constant introduction of dust into the repository air, the existence of amplification zones, and the presence of fungal growth on the repository walls and perhaps on other substrates not yet identified by us, the NARC conservators and researchers. A similar behavior for the genera *Aspergillus* and *Penicillium* was reported by Pyrri *et al.* [28].

Several species were isolated from the indoor air, which turned out to be ecologically abundant. They were *A. flavus, A. niger, A. terreus, A. versicolor, C. cladosporioides, C. gossypiicola, C. lignicola, C. oxysporum, C. sphaerospermum, P. chrysogenum, P. citrinum, P. commune* and *P. simplicissimum*; although other species of *Aspergillus, Cladosporium* and *Penicillium* were also detected with minor RF. It is noteworthy that the species *Cladosporium allii-porri, C. elatum, C. fulvum, C. minourae* and *P. corylophilum* were new findings for Cuban archives environments.

About the settled dust in the studied repositories, it should be noted that the values obtained in this research were higher than those reported by Awad *et al.* [11] in a study carried out in a museum in Cairo, Egypt, where they registered loads of 0.1-8.4 mg/m^2^/day in the different analyzed spaces, but in the repositories the dust burden was very low (0.1-1.2 mg/m^2^/day). However, the obtained values were much lower than those reported by Rodríguez [48] in a study carried out in the repository of the National Museum of Music in Cuba (64 mg/m^2^/day) a heritage institution located in Old Havana, very near the Port Avenue and the NARC. On the other hand, the fungal load values were also lower than those reported by Valdés *et al.* [55] in an investigation performed inside the Basilica of the San Francisco de Asís Convent (26.5 – 52.8 mg/m^2^/day), an institution that is also located very close to Port Avenue and the NARC and, consequently, it can be stated that the amount of dust collected from these repositories was low. In the statistical analysis carried out to compare the accumulated dust load between the two sites of the building (north and south), significant differences were found between the repositories on the first floor and the rest of the repositories, being R-14 the one that showed the highest values. This repository is one of the most exposed to the entry of dust through the ventilation ducts, since it is located on the south side of the building, the part that receives the greatest impact from outside pollution [40] and dust due to the existence of large avenues, groves, high vehicular and pedestrian traffic, etc. Although the dust accumulation within a built environment is the result of many factors (open/closed windows, ventilation system, human activities, green areas around the property, etc.) and not only the influence of the outdoor, in this study a marked influence was evidenced from the outdoor in the settled dust load, in agreement with previous reports [4, 13, 24, 56]. This may be due to the constant entry of dust through the natural ventilation system. Although a good part of the dust is trapped in the ventilation ducts, it is not always systematically removed from all of them. The ducts located at a height of 1.5 m are vacuumed once a month at the same time as the outside of the documents, the shelves and the floor, but those located at 4.5 m height are rarely cleaned, hence they represent important elements of the continuous entry of dust into the repositories.

In this study the fungal loads that were detected in dust were kept in lower ranges and/or similar to those reported by other authors. The fungal burden associated to the settled dust on selected books or archive materials in Polish libraries and one archive oscillated between 49 x 10^3^ - 450 x 10^3^ CFU g^−1^ of dust [14], while the fungal concentration obtained from the surfaces of books, shelves and furniture of two Egyptian libraries ranged between 0 - 6.3 x 10^5^ CFU g^−1^ [17]. However, fungal concentrations associated with deposited dust in an Egyptian museum ranged from ∼ 10^2^ to 10^4^ CFU g^−1^ [11]. The fungal concentrations detected in this study were markedly lower than those reported by OSHA [57], which establishes limit concentrations in the order of 10^6^ CFU g^−1^ of dust. It is known that many microorganisms are associated with dust [58, 59] and in archives and libraries where dust tends to be high, this phenomenon is more marked [4, 6, 14]. Therefore, the sanitation strategy of the repositories should be improved to ensure not only their cleanliness but also a convenient way to cleanse the ducts located at 4.5 m height. Frequent cleansing removes the accumulated dust, prevents the resuspension of all settled allergens, thus eliminating one of the basic sources of indoor fungal aerosol. These measures are part of the strategies that guarantee the preservation of the documentary collections in the archives.

In the collected dust, 14 taxa were detected, some of them such as *Alternaria, Aspergillus, Cladosporium, Penicillium* and *Zygosporium* were isolated from the dust, of the all naturally ventilated repositories, other were only detected in the dust collected in the repositories located on the south side of the building (R-4, R-14 and R-24) e.g. *Curvularia, Harposporium, Neurospora* and *Scolecobasidium*. Yeasts were detected in almost all repositories except in R-28 (located on the 2^nd^ floor). It should be noted that *Aspergillus* teleomorphs were detected only in the collected dust of repositories located in the southern part of the building. This may be due to the characteristics of the dust (water activity, pH, etc.) in that area, which could have guaranteed the viability and subsequent development of these teleomorph. *Aspergillus* teleomorphs in indoor dust has also been detected in previous studies [6, 16, 17].

In this case, *Aspergillus, Cladosporium* and *Penicillium* were again predominant, although *Alternaria* and *Zygosporium* also prevailed. These genera abound in the atmosphere of Havana and have been previously detected, although *Curvularia* and *Alternaria* are also among the most common genera in this environment [51, 60] and may have accumulated in the dust that penetrated the repositories and settled in the collectors.

*Aspergillus, Cladosporium* and *Penicillium* were previously detected as predominant genera in the dust of an Italian archive [6], in French archives [16] and an Egyptian museum [11]. In investigations made in Polish archives, libraries and museums, several species of these three genera were isolated among dust fungi by a method different from that used in this study [14, 48, 56, 61]. On the other hand, these genera were also predominant in household dust [7], although their characteristics may be different from those of an archive, library or museum.

The presence of *Harposporium, Neurospora* and *Scolecobasidium* genera in the collected dust of the ventilated repositories located to the south of the building, as well as *Humicola* genus detected only in the Map library dust (also located at south side) may have originated from the vegetation found in the adjacent park and in an area closely situated in this side of the building [62–64]. Fungal diversity in the dust could be influenced by the green areas and the total area of vegetation in the vicinity of the studied site [4].

About the species belonging to the predominant genera in the dust (*Aspergillus, Cladosporium* and *Penicillium*), it was shown that 48.1% of them were abundant according to their percentages of RF; the remaining species were classified as common (7.5%), frequent (22.2%), occasional (18.5%) and rare (3.7%). The distribution of the abundant species by genus showed that 50% of them corresponded to the *Aspergillus* genus, distinguishing *A. chevalieri, A. flavus* and *A. niger*, and 55.6% belonged to *Cladosporium*, standing out *C. cladosporioides* and *C. sphaerospermum*. It was also found that 37.5% of *Penicillium* species were abundant, highlighting *P. chrysogenum* and *P. citrinum*.

In this study, for the first time, the similarity (QS) of isolated taxa in the three analyzed niches (indoor air of the NARC repositories, outdoor air and settled dust) was analyzed. High QSs were obtained by comparing taxa detected in indoor air and outdoor air as well as between indoor air and settled dust. Hence, the contribution of the species belonging to the prevailing genera depended on the analyzed niche. The comparison of species between indoor and outdoor air showed a high QS for *Aspergillus* and *Cladosporium*, while for *Penicillium* it was low since species of this genus were only detected in the first sampling. However, the QSs obtained when comparing indoor air and dust were high (QS ≥ 0.6), particularly for the *Aspergillus* genus (QS = 0.8). These results showed that the outdoor air has an important influence on the repository's indoor air, mainly due to the existing natural cross-ventilation system and that the dust particles within the repositories (either because they penetrated from the outdoor or because they come from the ventilation ducts, or from walls with fungal growth, etc.) contribute significantly to the enrichment of airborne mycobiota indoors. Twelve of the detected species in this study were similar in the three analyzed niches and were *A. flavus, A. niger, A. oryzae, A. penicillioides, A. terreus, C. elatum, C. oxysporum, C. sphaerospermum, C. tenuissimum, P. aurantiogriseum, P. chrysogenum* and *P. citrinum.* Some of these species are recognized for their allergenic properties [22], while other species isolated in this work (e.g. *A. chevalieri, A. clavatus, A. flavipes, A. glaucus, P. brevicompactum, P. commune*, etc.) are considered opportunistic pathogens [65].

Many of the species detected both in the repositories indoor air and in the settled dust have been previously isolated from the outdoor air [52, 60, 66], suggesting that they are typical species of the Havana environment, although they have also been reported in countries of other world tropical and subtropical regions [42] characterized by high T and RH values, mainly the species of the genus *Aspergillus* [60, 67, 68]. It has even been reported that *Aspergillus* and *Cladosporium* correlate positively with high T and RH values [60, 69, 70], and that in particular RH contributes to spore dispersion and to maintain a high viability of its propagules in the air [60, 69].

On the other hand, in this study species belonging mainly to primary and secondary colonizers were also detected [11, 71], both in the repositories air and in the settled dust, demonstrating the risk that documentary collections run of being damaged by these fungi. In addition, poor air circulation may facilitate the deposition of airborne fungal propagules on documents, thus facilitating fungal growth on these materials. For this reason, some degradative tests were carried out on the isolated strains with the intention of learning their biodeteriogenic potential.

As it was shown, all strains isolated both in the air and in the settled dust were able to degrade to a greater or lesser extent the tested nutritional sources that are part of the components of paper and other documentary materials (cellulose, starch, gelatin), it is important to pay special attention to the preventive conservation measures fundamentally related to the hygiene of documents and repositories, with the guarantee that all documents have quality packaging that mitigates the harmful effect of dust and environmental fungi, as well as a good air circulation in the repositories since the T and RH cannot be controlled due to the existing natural ventilation system in these premises. Fungi are mostly mesophilic, acidophilus (pH 4 - 6) and grow well at relative humidity above 70%. Only if the temperature, water activity and acidity in the substrate are favorable, the fungal spores settled on the documents can germinate and grow abundantly; but the main limiting factor that determines the development of fungi in these materials is water, although some xerophilic/halophilic fungi have been associated with these materials [72]. It has been reported that species of *Aspergillus* (e.g., *A. flavus, A. niger, A. terreus*), *Cladosporium* (such as *C. cladosporioides, C. sphaerospermum*) and *Penicillium* (e.g., *P. chrysogenum* and *P. citrinum*) have cellulolytic, amylolytic and proteolytic activities, excrete acids and many of them also excrete pigments [17, 19, 20, 35, 47, 48, 51]; hence, they are species that have been linked with the documents' biodegradation [21, 25, 38]. Specifically, the aforementioned species and many others were capable of degrading cellulose, starch and gelatin. Among them, the following were distinguished by their high CEIs: *A. chevalieri, A. clavatus, A. ochraceus, A. oryzae, A. versicolor, C. basiinflatum, C. caryigenum, P. decumbens, P. griseofulvum* and *P. simplicissimum*. The species *A. ochraceus, A. oryzae, A. versicolor, C. herbarum, C. basiinflatum* and *P. simplicissimum* outstand for their high AEIs, while *A. glaucus, A. ochraceus, A. oryzae, C. basiinflatum, C. cladosporioides, C. oxysporum, P. decumbens, P. digitatum, P. oxalicum* and *P. simplicissimum* stood out for their high PEIs. But the most important aspect in the document's preservation is the number of degradative attributes that one fungal strain has [20] because it is indicative of its biodeteriogenic potential. However, it was shown in this investigation that most of the evaluated strains were positive to four or five of the analyzed physiological tests, showing that they are high-risk agents for the documentary collections preserved at the NARC. Similar results were previously reported [20, 26, 50].

The obtained results show the potential risk to which the documents conserved in these repositories are exposed. Hence, the importance of maintaining a systematic prevention strategy in the NARC where the hygiene-sanitary conditions of the repositories, furniture and documents are the most important aspect to take into account, hygiene actions that must always be carried out include the usage of appropriate personal protective equipment. The NARC has had a Preventive Conservation Plan for years that systematically executes and updates what has allowed it to preserve the documentary heritage it treasures in good condition despite the climatic conditions of the country. Just all of the research being done at NARC on airborne fungi, environmental mycological quality, and other environmental aspects contributes to the continual improvement of that Plan.

## MATERIALS AND METHODS

### Characteristics of the analyzed repositories

**Figure 3:**
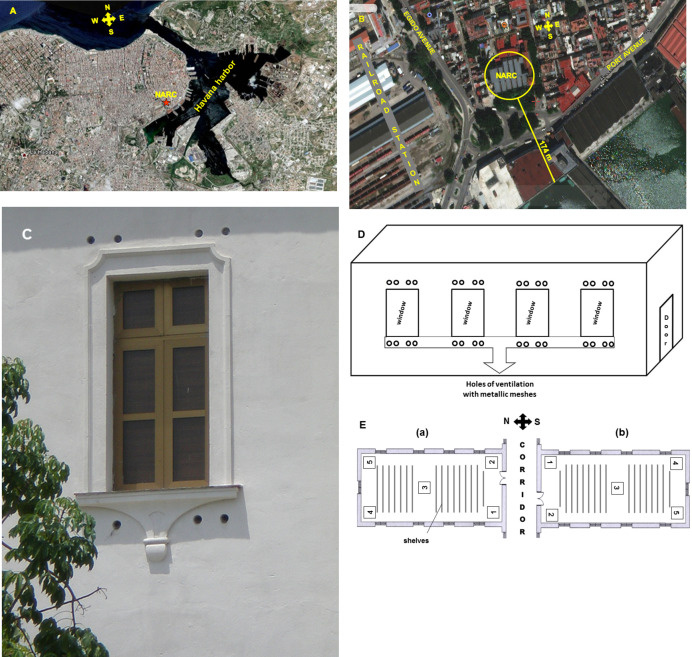
FIGURE 3: Satellite location of the National Archive of the Republic of Cuba (NARC) in the city of Havana (A), aerial view showing its proximity to the sea, as well as the avenues and the vegetation that surround the building (B), photo (C) and schematic representation (D) of natural ventilation system openings location in the repositories as well as the microbiological sampling points (E) in each repository analyzed (a, b). At position (a) or north side of the building are repositories R-8, R-20, R-28, and at position (b) or south part are repositories R-4, R-14, R-24.

The building of the NARC was constructed in the period of 1940 to 1944 in an old urban area of Havana city known as Old Havana, 174 m from Havana harbor (**Figure 3A** and **3B**). The NARC building is located in a highly polluted area of Havana [41]. Bordering its southern part there is a park with a lot of vegetation and a wide avenue (Port Avenue) that has a high pedestrian and vehicular traffic that generates a lot of environmental dust. Also, on its north, east and west sides it has a large number of houses that generate garbage and dirt that is deposited in garbage tanks until it is collected. In addition, it is very close to the Havana harbor, an electricity plant, the railroad station and other busy avenues.

The building has 30 repositories located on three floors. The majority of the repositories (28) have holes (10 cm diameter) that cross over the outer walls in an angle of 45° approximately, and they are protected by metallic meshes. These holes were made at different heights (1.5 and 4.5 m, approximately) to facilitate the inflow of outside air into the repositories and to secure a good natural cross-ventilation system (**Figure 3C** and **3D**). Also, NARC has two repositories with air-conditioning and one of them is the Map library.

For the selection of the repositories with natural cross ventilation, their distribution on the three floors of the building and their location on the north or south side were considered. Of the two air-conditioned repositories, the Map library was selected because it is the largest (T = 22 ± 2°C y RH = 50 ± 5%). The studied repositories were R-4 and R-8 (situated on the semi-basement), R-14 and R-20 (in the first floor), R-24 and R-28 (in the second floor), as well as the Map library (in the first floor, too). These repositories are characterized by their large size, their dimensions (length x width x height, m) being the following: R-4: 15.2 x 6.2 x 2.5; R-14, R-24 and Map library: 15.2 x 6.2 x 5; R-8: 25.3 x 6.2 x 2.5; R-14 and R-28: 25.3 x 6.2 x 5.

### Airborne fungal sampling

The number of points to be sampled was determined according to Sanchis [73] who reports a simple method based on the cube root of the volume of the premises (**Figure 3E**). According to this criterion, five points were sampled in triplicates.

In the environmental microbiological samplings of indoor and outdoor points a SAS biocollector (Super 100 ^TM^, Italy) was used with an air flow of 100 L min^−1^ for 2 min at a height of 1.5 m in vertical position at intervals of one hour between replicates. Two variants of the culture medium were used to guarantee the greatest possible fungal diversity and were Malt Extract Agar (MEA; Biocen, Cuba) at pH 5 [52] and MEA supplemented with NaCl (7.5%) [26, 32]. Then, the dishes were invert incubated for 5 to 7 days at 30°C. After the incubation, colony forming units were counted, which were them transformed into colony forming units per m^3^ (CFU m^−3^) following the instructions described in the equipment manual [74].

The indoor/outdoor (I/O) ratio was calculated according to Stryjakowska-Sekulska *et al.* [44], Awad *et al.* [11] and Karbowska-Berent *et al.* [14]. These authors reported that a good quality environment has an I/O ratio ≤ 1, and that an I/O ratio > 1 is a very strong indication of indoor sources of contamination.

### Settled dust and its fungal load

A passive method was used in sample the dry settled dust. For this, six months before the environmental microbiological sampling, dust collectors were located in the same five points where the environmental sampling was carried out. As these collectors were placed on the shelf, they were at a height of 3 m from the floor in the repositories with natural cross ventilation and at 2 m in the Map library. These collectors consisted of sterile 110 mm plastic Petri dishes (previously weighed) that were left open for six months after being placed at the sampling points. Thus, the dust was deposited on the surface of the open dishes in the same way that it does on the documents [75]. During the capture of the dust collectors, they were closed with their corresponding covers and were transported to the laboratory. Subsequently, they were placed in desiccators with silica gel and weighed every 24 h until the collectors reached constant weight.

The determination of the total load of settled dust was carried out according to the formula proposed by Oliva *et al.* [76], and the deposition rate was calculated as mg/m^2^/day.

Total dust load = (Pf - Pi) / A x t

Where: Pf- final dry weight of the Petri dish with powder, Pi- initial dry weight of the sterile Petri dish, A- area of the dish (m^2^), t- time (days)

For the microbiological sampling of the dust, 0.01 g of the settled dust was taken from each collector and 0.5 mL of sterile distilled water was added. Samples were allowed to stand for 1 h and each sample was randomly shaken well at intervals for 45 min. Then serial dilutions were made and seeded in depth in 110 mm plates with MEA + NaCl and MEA at pH 5. Dishes were invert incubated for 5 to 7 days at 30°C. After the incubation, the colony forming units were counted in order to determine the fungal concentration expressed in colony forming units per g of collected dust (CFU g^−1^).

CFU g^−1^ = (Number of total CFUs obtained x dilution) / 0.01 g of collected dust

### Identification of airborne and dustborne fungi

For the identification of fungal isolates cultural and morphological characteristics of the colonies, as well as the conidiophores and conidia structures were observed in a stereomicroscope (X14) and a clear field trinocular microscope (Olympus, Japan) at X40 and X100 coupled to a digital camera (Samsung, Korea). Different mycological key manuals were used [62, 77–85].

### Ecological approaches

Relative Density (RD) of fungal genera isolated from indoor air of each repository was conducted according to Smith [86], where:

RD = (number of colonies of one taxon / total number of colonies) x 100

Relative Frequency (RF) of the fungal species detected on indoor environments as well as the genera and species isolated from dust collected was determined according to Esquivel et al. [87], where:

RF = (times a genus is detected / total number of sampling realized) x 100

The ecological categories are classified as: Abundant (A) with RF = 100-81%, Common (C) with RF = 80-61%, Frequent (F) with RF = 60-41%, Occasional (O) with RF = 40-21%, Rare (R) with RF = 20-0% [32].

The Sørensen's coefficient of similarity (QS) was used to compare the similarities of obtained taxa among the three ecological niches (indoor air, outdoor air, and collected dust). The comparisons made were between the indoor air and the outdoor as well as between the indoor air and the collected dust [52].

QS = 2a/b + c

Where: a- is the number of common genera detected in the two environments that are comparing, b- the number of detected taxa only in indoor environment and c- the number of detected taxa only in the outdoor environment.

The QS values must range between 0 and 1. A value equal to 0 indicates that the taxa obtained in both compared environments are completely different and a value equal to 1 display that the taxa are identical [88].

### Determination semi-quantitative of the biodegradation potential of the isolated taxa

#### Determination of enzymatic index (EI)

To quantify the cellulolytic, amylolytic and proteolytic enzymatic index (EI), the following formula was used [26, 89, 90]:

EI = 1- Dc / Dca

Where: Dc is the colony diameter, Dca is the sum of Dc and the diameter of the hydrolysis zone. Values between 0.5 and 0.59 were classified as low EI, between 0.6 and 0.69 as moderate EI, and above 0.7 as high. Each determination was made in triplicate and averages are reported.

#### Cellulolytic enzymatic index (CEI)

The different strains of each species were inoculated in Petri dishes containing an agarized culture medium with a saline composition for one liter of: sodium nitrate 2 g, potassium phosphate 1 g, magnesium sulfate 0.5 g, ferrous sulfate 0.01 g, chloride potassium 0.5 g, yeast extract 0.5 g and 20 g of agar technical No. 1. As a carbon source, carboxymethylcellulose (CMC) at 1% was added and incubated at 30°C. After seven days, a solution of Congo red (0.05 g l^−1^) was added to each dish and was maintained by one hour, then the solution was decanted and NaCl at 1 mol l^−1^ was added for 10 min. Cellulolytic activity was evidenced by the formation of a white halo around the colony [26].

#### Amylolytic enzymatic index (AEI)

Each strain was inoculated in a Petri dish with a saline composition similar to the one previously used and starch was (1%) employed as the carbon source. After 7 days of incubation at 30°C, approximately 5 ml of Lugol's reagent were added to each culture dish, and the presence of a colorless zone around the colonies was taken as positive hydrolysis [20, 24, 26].

#### Proteolytic enzymatic index (PEI)

The strains were inoculated in dishes containing an agarized culture medium with a saline composition similar to that used previously, with gelatin as the carbon source (1%). The dishes were incubated at 30°C; the test reading was performed at 7 days of incubation with the addition of the Frazier reagent. A white precipitate around the colony (halo) was taken as the presence of non-hydrolyzed gelatin but a colorless halo indicated gelatin hydrolysis [26, 90, 91].

#### Determination of the acid excretion

A suspension of spores from each strain was seeded (0.1 ml) in a minimal liquid medium of identical composition to the one above, but with glucose at 1% as carbon source, 0.03% of phenol red and the pH adjusted to 7. The cultures were incubated at 30°C for 3 days. A change of color from red to yellow was indicative of the production of acids and the pH of the culture medium was measured using a pH meter (Pacitronic MV 870, USA) [20, 26].

#### Determination of extracellular pigments excretion

The strains were inoculated in tubes with slants containing an agarized culture medium with a saline composition similar to CMC medium but with glucose as the carbon source (1%). The tubes were incubated at 30°C during 7 days and excretion of diffusible pigments was observed in the culture medium of each tube. Also, the pigment excretion in the medium with CMC was taken into account [26].

### Statistical analysis

In the statistical processing of the data the Statgraphics Centurion XV program was used. Student's t test was used to compare the obtained values in the repositories located to the south and the north in the same floor of the building. A simple variance analysis (ANOVA-1) and Multiple Ranges test by the method of least square difference (LSD) were performed to compare the obtained data of the total dust loads and fungal concentrations. A p-value smaller or equal to 0.05 was considered statistically significant.

## Abbreviations:

AEI: – amylolytic EI,
CEI: – cellulolytic EI,
CFU: – colony forming unit,
EI: – enzymatic index,
I/O: – indoor/outdoor,
NARC: – National Archive of the Republic Cuba,
PEI: – proteolytic EI,
PNSM: – pigmented non-sporulating mycelia,
QS: – Sørensen's coefficient of similarity,
RD: – relative density,
RF: – relative frequency,
WNSM: – white non-sporulating mycelia.

## References

[1] Fröhlich-Nowoisky J, Kampf CJ, Weber B, Huffman JA, Pöhlker C, Andreae MO, Lang-Yona N, Burrows SM, Gunthe SS, Elbert W, Su H, Hoor P, Thines E, Hoffmann T, Després VR, Pöschl U (2016). Bioaerosols in the Earth system: Climate, health, and ecosystem interactions.. Atmos Res.

[2] De Nuntiis P, Palla F, Palla F, Barresi G (2017). Biotechnology and conservation of cultural heritage..

[3] Górny RL, Harkawy AS, Ławniczek-Wałczyk A, Karbowska-Berent J, Wlazło A, Niesler A, Gołofit-Szymczak M, Cyprowski M (2016). Exposure to culturable and total microbiota in cultural heritage conservation laboratories.. Int J Occup Med Environ Health.

[4] Pinzari F, Abdul-Wahab SA (2011). Sick Building Syndrome in Public Buildings and Workplaces..

[5] Schweitzer MD, Calzadilla AS, Salamo O, Sharifi A, Kumar N, Holt G, Campos M, Mirsaeidi M (2018). Lung health in era of climate change and dust storms.. Environ Res.

[6] Maggi O, Persiani AM, Gallo F, Valenti P, Pasquariello G (2000). Airborne fungal spores in dust present in archives: Proposal for a detection method, new for archival materials.. Aerobiologia.

[7] Rintala H, Pitkäranta M, Täubel M (2012). Microbial communities associated with house dust.. Adv Appl Microbiol.

[8] Meklin T, Haugland RA, Reponen T, Varma M, Lummus Z, Bernstein D, Wymer LJ, Vesper SJ (2004). Quantitative PCR analysis of house dust can reveal abnormal mold conditions.. J Environ Monit.

[9] Nevalainen A, Morawska L (2009). http://www.ilaqh.qut.edu.au/Misc/BIOLOGICAL_AGENTS_2009.pdf.

[10] Pasquarella C, Saccani E, Sansebastiano GE, Ugolotti M, Pasquariello G, Albertini R (2012). Proposal for a biological environmental monitoring approach to be used in libraries and archives.. Ann Agric Environ Med.

[11] Awad AHA, Saeed Y, Shakour AA, Abdellatif NM, Ibrahim YH, Elghanam M, Elwakeel F (2020). Indoor air fungal pollution of a historical museum, Egypt: A case study.. Aerobiologia.

[12] Aleksic B, Draghi M, Ritoux S, Bailly S, Lacroix M, Oswald IP, Bailly J-D, Robinec E (2017). Aerosolization of mycotoxins after growth of toxinogenic fungi on wallpaper.. Appl Environ Microbiol.

[13] Pinzari F, Gutarowska B, Joseph E (2021). Microorganisms in the Deterioration and Preservation of Cultural Heritage..

[14] Karbowska-Berent J, Górny RL, Strzelczyk AB, Wlazło A (2011). Airborne and dust borne microorganisms in selected Polish libraries and archives.. Build Environ.

[15] Skóra J, Gutarowska B, Gutarowska B (2016). A modern approach to biodeterioration assessment and the disinfection of historical book collections..

[16] Roussel S, Reboux G, Millon L, Parchas MD, Boudih S, Skana F, Delaforge M, Rakotonirainy MS (2012). Microbiological evaluation of ten French archives and link to occupational symptoms.. Indoor Air.

[17] Osman ME, Abdel-Hameed AA, Ibrahim HY, Yousef F, Abo-Elnasr FF, Saeed Y (2017). Air microbial contamination and factors affecting its occurrence in certain book libraries in Egypt.. Egypt J Bot.

[18] Pinheiro AC (2014). https://run.unl.pt/bitstream/10362/14890/1/Pinheiro_2014.pdf.

[19] Mallo A, Nitiu D, Elíades L, Saparrat M (2017). Fungal degradation of cellulosic materials used as support for cultural heritage.. Int J Conserv Sci.

[20] Borrego S, Molina A, Santana A (2017). Fungi in archive repositories environments and the deterioration of the graphics documents.. EC Microbiol.

[21] Michaelsen A, Piñar G, Pizari F (2010). Molecular and microscopical investigation of the microflora inhabiting a deteriorated Italian manuscript dated from the Thirteenth century.. Microb Ecol.

[22] Simon-Nobbe B, Denk U, Pöll V, Rid R, Breitenbach M (2008). The spectrum of fungal allergy.. Int Arch Allergy Immunol.

[23] Barnes CS, Alexis NE, Bernstein JA, Cohn JR, Demain JG, Horner E, Levetin E, Nel A, Phipatanakul W (2013). Climate change and our environment: The effect on respiratory and allergy disease.. J Allergy Clin Immunol Pract.

[24] Guiamet PS, Borrego S, Lavin P, Perdomo I, Gómez de Saravia S (2011). Biofouling and biodeterioration in material stored at Historical Archive of the Museum of La Plata, Argentine and at the National Archive of the Republic of Cuba.. Colloid Surface B.

[25] Lavin P, Gómez de Saravia S, Guiamet PS (2014). An environmental assessment of biodeterioration in document repositories.. Biofouling.

[26] Borrego S, Molina A (2020). Behavior of the cultivable airborne mycobiota in air-conditioned environments of three Havanan archives, Cuba.. J Atmospheric Sci Res.

[27] Pasquarella C, Balocco C, Saccani E, Capobianco E, Viani I, Veronesi L, Pavani F, Pasquariello G, Rotolo V, Palla F, Albertini R (2020). Biological and microclimatic monitoring for conservation of cultural heritage: A case study at the De Rossi room of the Palatina library in Parma.. Aerobiologia.

[28] Pyrri I, Tripyla E, Zalachori A, Chrysopoulou M, Parmakelis A, Kapsanaki-Gotsi A (2020). Fungal contaminants of indoor air in the National Library of Greece.. Aerobiologia.

[29] Borrego S, Perdomo I (2012). Aerobiological investigations inside repositories of the National Archive of the Republic of Cuba.. Aerobiologia.

[30] Borrego S, Molina A (2014). Comportamiento de la aeromicrobiota en dos depósitos del Archivo Nacional de la República de Cuba durante 7 años de estudio.. AUGMDOMUS.

[31] Molina A, Borrego S (2014). Análisis de la micobiota existente en el ambiente interior de la mapoteca del Archivo Nacional de la República de Cuba.. Bol Micol.

[32] Borrego S, Perdomo I (2016). Airborne microorganisms cultivable on naturally ventilated document repositories of the National Archive of Cuba.. Environ Sci Pollut Res.

[33] Borrego SF, Molina A (2018). Determination of viable allergenic fungi in the documents repository environment of the National Archive of Cuba.. Austin J Public Health Epidemiol.

[34] Borrego S, Perdomo I (2014). Caracterización de la micobiota aérea en dos depósitos del Archivo Nacional de la República de Cuba.. Rev Iberoam Micol.

[35] Borrego S, Guiamet P, Vivar I, Battistoni P (2018). Fungi involved in biodeterioration of documents in paper and effect on substrate.. Acta Microsc.

[36] Molina A, Borrego S (2015). El planero como barrera contra agentes biodeteriorantes de mapas y planos.. Ph investigación.

[37] Skóra J, Gutarowska B, Pielech-Przybylska K, Stępień L, Pietrzak K, Piotrowska M, Pietrowski P (2015). Assessment of microbiological contamination in the work environments of museums, archives and libraries.. Aerobiologia.

[38] Rahmawati SL, Zakaria L, Rahayu ES (2018). The diversity of indoor airborne molds growing in the university libraries in Indonesia.. Biodiversitas.

[39] Kadaifciler GD (2017). Bioaerosol assessment in the library of Istanbul University and fungal flora associated with paper deterioration.. Aerobiologia.

[40] Chen C, Zhao B, Zhang Y, Hopke PK, Mandin C (2021). Handbook of Indoor Air Quality..

[41] Cuesta-Santos O, González-Jaime Y, Sosa-Pérez C, López-Lee R, Bolufé-Torres J, Reyes-Hernández F (2019). La calidad del aire en La Habana. Actualidad.. Rev Cuba Meteorol.

[42] Chakraborty P, Gupta-Bhattacharya S, Chowdhury I, Majumdar M, Chanda S (2001). Differences in concentrations of allergenic pollens and spores at different heights on an agricultural farm in West Bengal, India.. Ann Agric Environ Med.

[43] Li L, Lei C, Liu Z-G (2010). Investigation of airborne fungi at different altitudes in Shenzhen University.. Natural Science.

[44] Stryjakowska-Sekulska M, Piotraszewska-Pająk A, Szyszka A, Nowicki M, Filipiak M (2007). Microbiological quality of indoor air in university rooms.. Pol J Environ Stud.

[45] Rojas TI, Martínez E, Aira M, Almaguer M (2008). Aeromicota de ambientes internos: Comparación de métodos de muestreo.. Bol Micol.

[46] Rodríguez JC, Rodríguez B, Borrego SF (2014). Evaluación de la calidad micológica ambiental del depósito de fondos documentales del Museo Nacional de la Música de Cuba en época de lluvia.. AUGMDOMUS.

[47] Rodríguez JC (2016). Evaluación aeromicrobiológica del depósito del Centro de Documentación del Museo Nacional de la Música de Cuba.. Ge-conservación.

[48] Rodríguez JC (2016). Microbiología aplicada: Una herramienta para la conservación del Patrimonio Cultural.. Conserv Patrim.

[49] Borrego S, Molina A, Abrante T (2020). Sampling and characterization of the environmental fungi in the Provincial Historic Archive of Pinar del Río, Cuba.. J Biomed Res Environ Sci.

[50] Borrego S, Molina A, Castro M (2021). Assessment of the airborne fungal communities in repositories of the Cuban Office of the Industrial Property: Their influence in the documentary heritage conservation and the personnel's health.. Rev Cub Cien Biol.

[51] Rojas TI, Aira MJ, Batista A, Cruz IL, González S (2012). Fungal biodeterioration in historic buildings of Havana (Cuba).. Grana.

[52] Sánchez KC, Almaguer M, Pérez I, Rojas TI, Aira MJ (2019). Diversidad fúngica en la atmósfera de La Habana (Cuba) durante tres períodos poco lluviosos.. Rev Int Contam Ambie.

[53] Mallo AC, Nitiu DS, Elíades LA, García M, Saparrat MCN (2020). Análisis de la carga fúngica en el aire de la sala “Fragmentos de Historia a Orillas del Nilo” y del exterior del Museo de La Plata, Argentina.. Ge-conservación.

[54] Pinheiro AC, Sequeira SO, Macedo MF (2019). Fungi in archives, libraries, and museums: a review on paper conservation and human health.. Crit Rev Microbiol.

[55] Valdés C, Corvo F, González E, Pérez J, Portilla C, Cuesta O (2007). Mecanismos de deterioro de la piedra caliza coralina estructural del Convento y Basílica Menor de San Francisco de Asís y ensayo de productos para su conservación.. Rev CENIC Cienc Quim.

[56] Niesler A, Górny RL, Wlazło A, Łudzeń-Izbińska B, Ławniczek-Wałczyk A, Gołofit-Szymczak M, Meres Z, Kasznia-Kocot J, Harkawy A, Lis DO, Anczyk E (2010). Microbial contamination of storerooms at the Auschwitz-Birkenau Museum.. Aerobiologia.

[57] OSHA (Occupational Safety and Health Administration) (2002). OSHA Technical Manual, Section III, Ch. 2.

[58] Alghamdi MA, Shamy M, Redal MA, Khder M, Awad AH, Elserougy S (2014). Microorganisms associated particulate matter: A preliminary study.. Sci Total Environ.

[59] Stamatelopoulou A, Pyrri I, Asimakopoulos DN, Maggos T (2020). Indoor air quality and dustborne biocontaminants in bedrooms of toddlers in Athens, Greece.. Build Environ.

[60] Almaguer M, Díaz L, Fernández-González M, Salas S (2021). Assessment of airborne *Curvularia* propagules in the atmosphere of Havana, Cuba.. Aerobiologia.

[61] Harkawy A, Górny RL, Ogierman L, Wlazło A, Ławniczek-Wałczyk A, Niesler A (2011). Bioaerosol assessment in naturally ventilated historical library building with restricted personnel access.. Ann Agric Environ Med.

[62] Domsch KH, Gams W, Anders TH (1980).

[63] Cai KZ, Liu JL, Liu W, Wang BB, Xu Q, Sun LJ, Chen MY, Zhao MW, Wu JY (2016). Screening of different sample types associated with sheep and cattle for the presence of nematophagous fungi in China.. J Basic Microbiol.

[64] Piontelli E, Díaz MC (2004). El entorno humano y la relevancia biológica de las especies de *Neurospora*: Consideraciones en Micología Médica.. Bol Micol.

[65] de Hoog GS, Guarro G, Gene J, Figueras MJ (2000).

[66] Almaguer M, Rojas TI (2013). Aeromicota viable de la atmósfera de La Habana, Cuba.. Nova Acta Cient Compostelana (Bioloxía).

[67] Piontelli E (2008). Aportes morfotaxonómicos en el género *Aspergillus* Link: Claves para las especies ambientales y clínicas más comunes.. Bol Micol.

[68] Egbuta MA, Mwanza M, Babalola OO (2016). A review of the ubiquity of ascomycetes filamentous fungi in relation to their economic and medical importance.. Advances in Microbiology.

[69] Abdel Hameed AA, Khoder MI, Ibrahim YH, Saeed Y, Osman ME, Ghanem S (2012). Study on some factors affecting survivability of airborne fungi.. Sci Total Environ.

[70] Almaguer M, Aira MJ, Rodríguez-Rajo FJ, Rojas TI (2014). Temporal dynamics of airborne fungi in Havana (Cuba) during dry and rainy seasons: Influence of meteorological parameters.. Int J Biometeorol.

[71] Górny RL (2004). Filamentous microorganisms and their fragments in indoor air.. Ann Agric Environ Med.

[72] Micheluz A, Manente S, Tigini V, Prigione V, Pinzari F, Ravagnan G, Varese G (2015). The extreme environment of a library: Xerophilic fungi inhabiting indoor niches.. Int Biodeterior Biodegr.

[73] Sanchis J (2002). Los nueve parámetros más críticos en el muestreo biológico del aire.. Rev Tecn Lab.

[74] SAS Super 100™ (2001).

[75] Corvo F, Torrens AD, Martín Y, González E, Pérez J, Valdés C, Castañeda A, Portilla A (2008). Corrosión atmosférica del acero en interiores. Sus particularidades en el clima tropical de Cuba.. Rev de Metal.

[76] Oliva P, García K, Cortez R, Dávila R, Alfaro MR, Duke V (2001).

[77] Ellis MB (1976).

[78] Klich MA, Pitt JI (1994).

[79] Barnett HL, Hunter BB (1998).

[80] Pitt JI (2000).

[81] Varga J, Frisvad JC, Kocsubé S, Brankovics B, Tóth B, Szigeti G, Samson RA (2011). New and revisited species in *Aspergillus* section *Nigri*.. Stud Mycol.

[82] Varga J, Frisvad JC, Samson RA (2011). Two new aflatoxin producing species, and an overview of *Aspergillus* section *Flavi*.. Stud Mycol.

[83] Samson RA, Peterson SW, Frisvad JC, Varga J (2011). New species in *Aspergillus* section *Terrei*.. Stud Mycol.

[84] Bensch K, Groenewald JZ, Dijksterhuis J, Starink-Willemse M, Andersen B, Summerell BA, Shin H-D, Dugan FM, Schroers H-J, Braun U, Crous PW (2010). Species and ecological diversity within the *Cladosporium cladosporioides* complex (*Davidiellaceae, Capnodiales*).. Stud Mycol.

[85] Bensch K, Braun U, Groenewald JZ, Crous PW (2012). The genus *Cladosporium*.. Stud Mycol.

[86] Smith G (1980).

[87] Esquivel PP, Mangiaterra M, Giusiano G, Sosa MA (2003). Microhongos anemófilos en ambientes abiertos de dos ciudades del nordeste rgentine.. Bol Micol.

[88] Moreno CE (2001). Métodos para medir la biodiversidad.. M&T-Manuales y Tesis SEA, Zaragoza..

[89] Sterflinger K (2010). Fungi: Their role in deterioration of cultural heritage.. Fungal Biol Rev.

[90] Molina A, Borrego SF, Ortega BD (2017). Potencialidades biodeteriorantes y patogénicas de hongos anemófilos ambientales frecuentes en ambiente de archivos y museos cubanos.. Rev CENIC Cienc Biol.

[91] Iwatzu T (1984). A new species of *Cladosporium* from Japan.. Mycotaxon.

